# Supplementary material for: The Work and Social Adjustment Scale (WSAS): An investigation of reliability, validity, and associations with clinical characteristics in psychiatric outpatients
Source: PLoS One. 2024 Oct 10;19(10):e0311420. doi: 10.1371/journal.pone.0311420 (PMC11466382; doi:10.1371/journal.pone.0311420)
Supplement: S1 Table — (DOCX) [file pone.0311420.s001.docx]

**S1 Table**. Factor loadings for WSAS when combining the two subsamples

(50% *n_1_* and 50% *n_2_*)

| WSAS Items. Impaired: | *n_3_* |
| --- | --- |
| 1. Ability to work | 0.64 |
| 2. Home management | 0.74 |
| 3. Social leisure activities | 0.74 |
| 4. Private leisure activities | 0.68 |
| 5. Close relationships | 0.65 |

*Note*. WSAS = The Work and Social Adjustment Scale; *n_3_* = 1786.
