# Supplementary material for: The Work and Social Adjustment Scale (WSAS): An investigation of reliability, validity, and associations with clinical characteristics in psychiatric outpatients
Source: PLoS One. 2024 Oct 10;19(10):e0311420. doi: 10.1371/journal.pone.0311420 (PMC11466382; doi:10.1371/journal.pone.0311420)
Supplement: S2 Table — (DOCX) [file pone.0311420.s002.docx]

**S2 Table.** CFA results for WSAS based on subsample 4 (50% *n_1_* and 50% *n_2_)*

| Model | *χ*^2^ | RMSEA  [90% CI] | TLI | CFI | SRMR |
| --- | --- | --- | --- | --- | --- |
| Model | 124.36* | .12 [.10 – .14] | .91 | .95 | .04 |
| Model 2 | 18.49* | .05 [.03 – .07] | .99 | .99 | .05 |

*Note*. WSAS = The Work and Social Adjustment Scale; *n_4_* = 1787; * *p* <.001; RMSEA = Root Mean Square Error of Approximation; TLI = Tucker-Lewis Index; CFI = The Comparative Fit Index. Model 2 includes a correlation between components 3 and 5.
