# Supplementary material for: The Work and Social Adjustment Scale (WSAS): An investigation of reliability, validity, and associations with clinical characteristics in psychiatric outpatients
Source: PLoS One. 2024 Oct 10;19(10):e0311420. doi: 10.1371/journal.pone.0311420 (PMC11466382; doi:10.1371/journal.pone.0311420)
Supplement: Attachment — Submitted filename: Rebuttal letter3.1.docx [file pone.0311420.s003.docx]

**Rebuttal letter**

PONE-D-23-27409R1

PLOS ONE

The Work and Social Adjustment Scale (WSAS): An investigation of reliability, validity, and associations with clinical characteristics in psychiatric outpatients

Dear Editor, Dr. Ilias Mahmud,

We appreciate your feedback on our submitted manuscript titled “The Work and Social Adjustment Scale (WSAS): An investigation of reliability, validity, and associations with clinical characteristics in psychiatric outpatients “

We would like to express our sincere appreciation for the diligent work you and the editorial team have invested in reviewing our manuscript. Your valuable feedback has been carefully considered, and we have made the necessary revisions to address the comments provided in this point-by-point letter.

Best regards

On behalf of all authors,

Jakob Lundqvist

Journal Requirements:

When submitting your revision, we need you to address these additional requirements.

1. Please ensure that your manuscript meets PLOS ONE's style requirements, including those for file naming.

**Reply**: We have ensured the manuscript's adherence to PLOS ONE's style requirements.

2. Note from Emily Chenette, Editor in Chief of PLOS ONE, and Iain Hrynaszkiewicz, Director of Open Research Solutions at PLOS: Did you know that depositing data in a repository is associated with up to a 25% citation advantage (<https://doi.org/10.1371/journal.pone.0230416>)? If you’ve not already done so, consider depositing your raw data in a repository to ensure your work is read, appreciated and cited by the largest possible audience. You’ll also earn an Accessible Data icon on your published paper if you deposit your data in any participating repository (<https://plos.org/open-science/open-data/#accessible-data>).

**Reply**: Thank you for the information. All WSAS data have been de-identified and deposited openly (https://doi.org/10.3886/E198102V1).

3. We note that the grant information you provided in the ‘Funding Information’ and ‘Financial Disclosure’ sections do not match. When you resubmit, please ensure that you provide the correct grant numbers for the awards you received for your study in the ‘Funding Information’ section.

**Reply**: The Ph.D. position (stipend) of the first author (JL) was funded by NAV without a grant number attached to it. The Norwegian University of Science and Technology (NTNU) acts as an intermediary for the funds from the funding organization NAV. The university solely administers the funds by employing the first author as a Ph.D. candidate. The funding organization has no role in the study design, data collection and analysis, decision to publish, or preparation of the manuscript. None of the authors are affiliated with the funding organization.

4. Thank you for stating the following financial disclosure: 
"This work was funded by the Norwegian University of Science and Technology and the Norwegian Labour and Welfare Administration (NAV) [www.nav.no](http://www.nav.no). The funding resulted in a PhD candidate position for JL. The funders had no role in study design, data collection and analysis, decision to publish, or preparation of the manuscript. "

We note that one or more of the authors is affiliated with the funding organization, indicating the funder may have had some role in the design, data collection, analysis or preparation of your manuscript for publication; in other words, the funder played an indirect role through the participation of the co-authors. If the funding organization did not play a role in the study design, data collection and analysis, decision to publish, or preparation of the manuscript and only provided financial support in the form of authors' salaries and/or research materials, please do the following:

a. Review your statements relating to the author contributions, and ensure you have specifically and accurately indicated the role(s) that these authors had in your study. These amendments should be made in the online form.

b. Confirm in your cover letter that you agree with the following statement, and we will change the online submission form on your behalf: 
“The funder provided support in the form of salaries for authors [insert relevant initials], but did not have any additional role in the study design, data collection and analysis, decision to publish, or preparation of the manuscript. The specific roles of these authors are articulated in the ‘author contributions’ section.

**Reply**:

*a. The author contribution section is correct.*

*b. This statement has been confirmed in the cover letter.*

5. We note that you have indicated that there are restrictions to data sharing for this study. PLOS only allows data to be available upon request if there are legal or ethical restrictions on sharing data publicly. For more information on unacceptable data access restrictions, please see <http://journals.plos.org/plosone/s/data-availability#loc-unacceptable-data-access-restrictions>.

Before we proceed with your manuscript, please address the following prompts:

a) If there are ethical or legal restrictions on sharing a de-identified data set, please explain them in detail (e.g., data contain potentially identifying or sensitive patient information, data are owned by a third-party organization, etc.) and who has imposed them (e.g., a Research Ethics Committee or Institutional Review Board, etc.). Please also provide contact information for a data access committee, ethics committee, or other institutional body to which data requests may be sent.

b) If there are no restrictions, please upload the minimal anonymized data set necessary to replicate your study findings to a stable, public repository and provide us with the relevant URLs, DOIs, or accession numbers. For a list of recommended repositories, please see
<https://journals.plos.org/plosone/s/recommended-repositories>. You also have the option of uploading the data as Supporting Information files, but we would recommend depositing data directly to a data repository if possible.

We will update your Data Availability statement on your behalf to reflect the information you provide.

**Reply**: All WSAS data have been de-identified and openly uploaded to OpenICPSR (https://doi.org/10.3886/E198102V1). The deposited data exclude demographic information, in adherence to the ethical approval granted by the Research Ethics Committee (REK). We are constrained from sharing demographic details as it involves potentially identifiable and sensitive patient information. Moreover, the patients have not provided consent for the open sharing of such data.

6. Your ethics statement should only appear in the Methods section of your manuscript. If your ethics statement is written in any section besides the Methods, please move it to the Methods section and delete it from any other section. Please ensure that your ethics statement is included in your manuscript, as the ethics statement entered into the online submission form will not be published alongside your manuscript.

**Reply**: We have moved the ethics statement to the Methods section and deleted it from the acknowledgment section.

**Reviewer #1:**

1. Overall impression. The study brings to attention important data that interest many spheres of psychology and psychotherapy. Although the study shows differences in the WSAS between different subgroups, nevertheless, for scale validation, it is important to also measure the invariance between the groups (the size of at least two groups would allow this).

**Reply**: Thank you for your valuable feedback on our manuscript. We are grateful for the positive feedback on the study's relevance to psychology and psychotherapy.

We have included MI-testing across age, sick leave, and sex. In our analysis, WSAS demonstrated metric invariance, but scalar non-invariance, thus only partial invariance was found. This finding corroborates with Pedersen et al’s study. We have added the MI-testing to the manuscript under the Statistical Analysis section on page 9 and Results on page 12.

We found that invariance was mainly due to item 5, which was pointed out previously in the manuscript: men experience significantly higher impairment with close relationships. Therefore, it can be assumed, that males report higher impairment due to close relationships, regardless of WSAS score. Since partial invariance was due to a minority of parameters causing misfits, WSAS can still be accepted as an instrument measuring the same construct across e.g., genders.

2. Is it possible to reproduce the web-based portal name?

**Reply**: The revised manuscript mentions that the web-based portal name was checkware.no.

3. Participants and procedure: A table presenting the characteristics of the participants would be welcome.

**Reply**: This is now provided in Table 3 on page 23.

**Reviewer #2:** Dr. Roghieh Nooripour

The topic is fascinating, but a thorough restructuring of the content is necessary. I kindly request the esteemed author to revise the entire article with a deeper and more coherent perspective, taking into consideration the suggested changes. Afterward, please resubmit it for further review. I believe this feedback will be valuable in improving the quality of your work.

**Reply**: Thank you for providing helpful comments, which we believe have improved the manuscript.

**Abstract:**
1. The objective could be more specific about how the study contributes to existing knowledge or addresses a gap. For example, is this the first time the WSAS is being validated in a Norwegian setting or in these specific mental health conditions?

**Reply**: Changes have been made to the abstract (page 2) to highlight that the study is the first to investigate the validity and reliability of WSAS in a Norwegian routine care setting.

2. results could be enriched with more specific data or statistics. For instance, mentioning the value of composite reliability could add more credibility to your claim of high internal consistency. Also, quantifying the effect sizes when discussing the largest impairments could make your findings more impactful.

**Reply**: We have enriched the results section by including specific data, including the value of composite reliability and effect size.

3. Suggest areas where further research could build upon your findings. This shows a forward-thinking approach and situates your study within the broader research context.

**Reply**: We have revised the discussion and added suggested research areas for the future.

**Introduction**:

1. it could be more concise. Consider summarizing some of the background information to keep the focus on the main objectives of your study.

**Reply**: We have undertaken restructuring and clarifications at the end of the introduction, incorporating an informative opening, and summarizing the key points that underline the significance of the study.

2. the specific problem your study addresses could be stated more clearly. Emphasize the gap in research or practice your study aims to fill.

**Reply**: The two last parts of the introduction have been refined for conciseness, with a clearer statement of the specific problem our study addresses. Emphasis has been placed on highlighting the research gap or practical need that our study aims to fill. See the end of page 5 until the end of the introduction section.

3. Clearly state the primary objectives and hypotheses at the end of the introduction. This will provide a clear transition from the background to what your study specifically aims to achieve.

**Reply**: In response to your recommendation, we have revised the introduction to include a concise statement outlining the primary objectives and hypotheses of our study. Specifically, we have highlighted the central focus by stating: *"With an expectation of robust psychometric properties for WSAS within the Norwegian routine mental health care setting, our objective is to explore its correlation with sick leave status."*

4. Highlight the relevance of your study to current clinical practices or policy implications in Norway’s mental health care. This will underscore the importance of your research.

**Reply**: We have carefully considered your suggestion to highlight the relevance of our study to current clinical practices in Norway’s mental health care. This has been added to the end of the introduction on page 6.

5. Provide a brief rationale for choosing your methodology, particularly why WSAS was selected for this study and its relevance in the Norwegian healthcare context.

**Reply**: We have included a brief rationale for the chosen methodology of WSAS in the Norwegian healthcare context. It’s added under the measurement section on page 8: ***“****Previously, the Global Assessment of Functioning (GAF) scale was utilized in this psychiatric outpatient clinic; however, it was discontinued due to its unreliability in routine clinical settings [39]. WSAS was selected for clinical use based on its recommendation in clinical guidelines as a dependable instrument for evaluating disability or impairment arising from mental or physical health conditions [31]. The brevity, simplicity, and rapid completion time of WSAS (M = 1.5 min., SD = 1.3) have made it a recommended choice for both assessment and treatment evaluation [16,33].”*

6. Integrate studies more seamlessly into the narrative can enhance readability. Instead of listing studies, integrate them into a cohesive argument that builds towards your study's rationale.

**Reply**: In response to your suggestion, we have revised the introduction to better integrate previous studies into the narrative. Rather than listing studies, we have woven them into a more cohesive argument that builds toward our study's rationale. We believe this adjustment enhances the overall readability of the report and strengthens the flow of the narrative. The changes can be seen on pages 4 and 5.

7. Emphasize how your study adds to the existing literature. If your research addresses a limitation or gap not previously covered, make this clear to the reader.

**Reply**: Thank you for underscoring the significance of emphasizing our study's contribution to existing literature. We have clarified this study's unique contribution in the final part of the introduction on page 6.

**Method**:

1. Clarify why specific patient groups were excluded (e.g., those with OCD, schizophrenia, etc.). Explain how these exclusions might impact the generalizability of your findings.

**Reply**: We have no data on specific patient groups such as those with schizophrenia as they received treatment at other specialized units. Their unique treatment settings and interventions differ from the routine mental health care focused on in this study. Our study aimed to investigate the WSAS among individuals with common mental health disorders, enhancing its applicability to routine mental health care scenarios in Norway. We clarified this on page 7 and added a sentence under limitations on page 18: *“Furthermore, the applicability of the findings is restricted to general outpatient clinics that offer routine care and does not extend to specialized units.”*

2. Mention any ethical approvals obtained for the study, especially since it involves human subjects.

**Reply**: Information regarding the ethical approvals obtained for this study has been moved to the method section.

3. While you have explained WSAS well, briefly discuss its historical validity and reliability to establish its credibility as a measurement tool.

**Reply**: We appreciate your suggestion regarding a brief discussion on the historical validity and reliability of WSAS. We would like to highlight that comprehensive information on previous research and validation of WSAS is provided on page 5 in the introduction section.

4. Provide a rationale for choosing EFA and CFA, and explain why these methods are appropriate for your study.

**Reply**: We have included an additional sentence under Statistical Analyses on page 10:

“*This dual-method approach serves both to explore (EFA) and to confirm (CFA) the factor structure in two subsamples across diverse routine mental health care settings.* *It is common procedure if a measure is tested in a new setting [40].”*

5. Elaborate on the strategy for handling missing data beyond just removing non-responders. This is important for the validity of your results.

**Reply**: We conducted a Missing Completely At Random (MCAR) Little’s test, confirming the randomness of missing data patterns. It’s been added to page 9.

6. While you have listed various fit indices, explain what each one indicates and why it is relevant to your analysis.

**Reply**: We have incorporated a sentence on page 10: “*RMSEA, sensitive to model misspecification, measures the disparity between observed data and the model. SRMR measures the average standardized difference between observed and predicted correlations, while CFI and TLI assess the model's replication of the observed covariance structure [40].”*

7. When discussing group differences, specify how you controlled for potential confounders (if applicable).

**Reply**: We opted for an exploratory design for this study. In this exploratory context, we have limited ourselves to ANOVA. Since we are examining at the item level, it entails an increased risk of type 1 error with an increasing number of tests, for which we have not controlled for confounders. We have chosen not to do additional analyses in this study to prevent introducing excessive complexity. We have clarified the study's methodology under Statistical Analysis on page 9: *“This study employed an exploratory cross-sectional design to assess the reliability and validity of WSAS across all its items in Norwegian routine mental health care, while also examining variations in functional impairment based on sick leave status, psychiatric diagnosis, and sex.”*

**Discussion**

1. RMSEA value indicated a poor model fit, yet the SRMR value suggested a close-fitting model. Discuss why there is this discrepancy and what it implies for the validity of your model

**Reply**: The correlation between items 3 and 5 contributed to the RMSEA value in model 1, as explained on page 11*.* As previously mentioned in this letter, we have included a sentence on page 10: “*RMSEA, sensitive to model misspecification, measures the disparity between observed data and the model. SRMR measures the average standardized difference between observed and predicted correlations, while CFI and TLI assess the model's replication of the observed covariance structure.”* Additionally, we have incorporated a section discussing these results on page 14. “*The observed RMSEA value, sensitive to model complexity, suggested a poor fit for the unadjusted model, a discrepancy that can be traced back to error variance between items 3 and 5, influencing the RMSEA. However, in the case of Model 2, adjustments were made, resulting in an acceptable model fit. This modification aligns seamlessly with the theoretical perspective, particularly considering that items 3 and 5 explicitly address the impairment of social relatedness.”*

2. Compare reliability coefficients with those reported in previous studies, if available.

**Reply**: The findings from previous studies have been included in the discussion, see page 14.

3. Begin by explicitly linking your discussion to the study's objectives and key findings. This establishes a clear framework for your analysis.

**Reply**: We have revised the intro of the discussion section to explicitly connect our analysis with the expectation of robust psychometric properties. See page 13.

4. Elaborate on the implications of the one-factor model and good internal consistency for using WSAS in routine mental health care. Discuss how these findings contribute to the existing knowledge.

**Reply**: We have described how the WSAS functions for our sample and its applicability across sex and diagnosis, thus enhancing its utility as a valuable assessment tool. See page 15.

5. Explore the reasons behind the observed differences in functional impairment based on sick leave status, psychiatric diagnoses, and sex. Discuss how these findings align or contrast with existing research.

**Reply**: We have engaged in a discussion linking observed differences in functional impairment to previous research and anticipated outcomes. See page 15.

6. Compare your results with previous studies more explicitly. Where your findings agree or differ, discuss possible reasons for these similarities or discrepancies.

**Reply**: A discussion regarding possible reasons for similarities and discrepancies has been added to the discussion on page 15.

7. practitioners. How can these results inform clinical practice, especially in the context of assessing and addressing functional impairment?

**Reply**: We have addressed the practical implications of this study for mental health practitioners on page 19. This includes incorporating WSAS into routine assessment as a complementary tool alongside symptom assessment.

8. While you've identified some limitations, consider discussing how these might affect the interpretation of your results and the generalizability of your findings.

**Reply**: We have incorporated two sentences declaring that the design may limit the generalizability of the results on page 18.

9. Suggest specific areas for future research based on your findings and limitations. This could include longitudinal studies, the use of structured clinical interviews, or exploring functional impairment in other patient groups.

**Reply**: We have clarified recommendations for future research based on the limitations observed in this study. These can be found on page 19.

10. If relevant, discuss how cultural or societal factors might influence the reported differences in functional impairment, especially in the context of gender and mental health.

**Reply**: We have added a discussion of cultural factors regarding differences in impairment between genders at the end of page 15.

11. Offer specific recommendations for mental health policy or practice based on your findings. How might routine assessments be improved?

**Reply**: We have included recommendations for mental health practices on page 19.

12. Conclude the discussion by summarizing the main points, emphasizing the significance of your study, and restating its contribution to the field.

**Reply**: A summary of the main points, the significance of the finding, and practical implications have been added to the conclusion section on page 19.
