# Supplementary material for: The Work and Social Adjustment Scale (WSAS): An investigation of reliability, validity, and associations with clinical characteristics in psychiatric outpatients
Source: PLoS One. 2024 Oct 10;19(10):e0311420. doi: 10.1371/journal.pone.0311420 (PMC11466382; doi:10.1371/journal.pone.0311420)
Supplement: Attachment — Submitted filename: WSAS Rebuttal letter_aR2.docx [file pone.0311420.s004.docx]

**Rebuttal letter**

2024-09-03

Dr. Ilias Mahmud

Editor

PLOS ONE

Ref: PONE-D-23-27409R1: The Work and Social Adjustment Scale (WSAS): An investigation of reliability, validity, and associations with clinical characteristics in psychiatric outpatients

Dear Editor,

We appreciate your feedback on our manuscript. We sincerely appreciate the thorough review and valuable feedback. We have carefully considered the comments and made the necessary revisions.

Journal Requirements:

1. Please review your reference list to ensure that it is complete and correct. If you have cited papers that have been retracted, please include the rationale for doing so in the manuscript text, or remove these references and replace them with relevant current references. Any changes to the reference list should be mentioned in the rebuttal letter that accompanies your revised manuscript. If you need to cite a retracted article, indicate the article’s retracted status in the References list and also include a citation and full reference for the retraction notice.
***Reply***: We have reviewed the reference list to ensure it is complete and accurate.

**Reviewer #1:** I consider that the manuscript has been perfected and that the authors have addressed all the requirements.

**Reviewer #2:** The revisions to your paper have been diligently implemented, resulting in a fully corrected and now acceptable manuscript. We extend our gratitude for your dedicated efforts and collaborative approach in elevating the overall quality of your article.
We wish you continued success in your forthcoming research and writing endeavors.

***Reply to R1 and R2***: Thank you for your valuable feedback on our manuscript. We are grateful for the positive feedback.

**Reviewer #3:** The authors are commended for examining the psychometric properties of the scale in different cultures, languages, and settings in which the scale was originally developed as this important issue is routinely neglected.

***Reply***: Thank you for providing helpful comments, which we believe have improved the manuscript. Thank you for your positive feedback.

**1. Abstract**. It is unclear why the authors chose to do the EFA on 1/3 and CFA on 2/3 or sample? Why not use ½ of the sample for EFA and the other ½ for the CFA as is the common practice? The sample size is large enough that I am not concerned about the results being negatively impacted due to think, but this is more of a reflection to consider for future investigations.

***Reply***: Thank you for your suggestion. As we had data from two different clinics, we chose to do EFA on one and CFA on the other. However, we agree, that combining them could be a viable option. Therefore, we have conducted additional analyses as suggested by the reviewer.

In the methods section we have added that: “To further validate the robustness of the findings, supplementary analyses were conducted. Subsamples *n1* and *n2* were combined and then split into two (to represent the full spectrum of mental health issues). An EFA was then performed using half the sample (*n*3) and CFA on the other (*n*4).”

The following has been added to the results section regarding EFA:” Using EFA when combining subsamples 1 and 2 revealed near identical results (see supplementary Table S1). The KMO test was .81 for sample *n3*, and an orthogonal varimax rotation indicated a 1-factor solution with an eigenvalue of 2.39. When rotated, Factor 1 explained 48% of the variance of the total sample with factor loadings ranging from .64 to .74. The results from comparing scree plots and eigenvalues (i.e., eigenvalues > 1.00) suggested that the one-factor solution was a better fit for the data than the two- and three-factor solutions.”

The following was added for the CFA:” CFA was also used for modeling the unifactory solution (model 1) in subsample 4 (*n4* = 1787). The results are summarized in supplementary table S2 and were nearly identical to the original analysis. The RMSEA value indicated a poor model fit to the data (RMSEA = .12) and the SRMR value suggested an indicative of a close-fitting model (SRMR = .04), resulting in unsatisfactory fit statistics in *n4*. As with the original analysis, correlated residuals were observed for Items 3 and 5 (social relatedness) which were added to Model 2. Model 2 yielded an acceptable model fit to the data *χ*^2^**(4, *N* = 1787) _=_** 18.49, *p* <.001**,** RMSEA = .05, CFI = .99, TLI = .99, SRMR = .05. All factor loadings were positive and substantial (*p* < .001), with values ranging between *r* = .36 and .56.”

Therefore, these supplemental analyses supported the original one-factor solution, reinforcing the stability and robustness of our findings in clinical settings.

**2.** Pg 4. It would be helpful to flesh out the paragraph on differences in impairment across disorders. Does the literature describe reasons for the greater impairment in some groups (e.g., personality disorder) than others? How does comorbidity or disorder severity impact these patterns?

***Reply***: The following has been added to the introduction:*”* Differences in impairment between disorders could be due to both physical and psychological comorbidity, severity, and chronicity, but it is also important to note that the use of different instruments for measuring impairment could affect the results [14,15].”

**3.** Pg 9, setting section. Given the differences in the two sample (sample 1 – mild symptoms, sample two – moderate to severe symptoms) is seems that combining the samples and then conducting the EFA on ½ of the full sample and the other ½ of the combined sample would be a better approach for conducting the EFA and CFA to allow for more variation in symptom level across that EFA and CFA rather than truncating mild symptoms to EFA and moderate to severe in the CFA. Some rationale for the approach the authors used is needed.

***Reply***: Supplemental analyses have been added, incorporating the full spectrum of mental health issues from mild to severe. These analyses support the original one-factor solution and are detailed in Tables S1 and S2.

**4.** **Methods / analyses** – Do the authors know the reason for why people were on sick leave? Was the leave related to mental health problems? What about physical health issues? Are the authors able to report on level of physical health issues in the sick leave group. If not, this should be listed as a key confound variable as differences in physical health problems in the sick leave group may be driving the results more than the specific mental health diagnoses.

***Reply***: In the methods section we have added the following: “Unpublished data from the two clinics suggest that the most common reasons for being on sick leave at the start of treatment were psychological problems (72.2%), musculoskeletal disorders (7.1%), and neurological disorders (4%). The analyses did not explore the impact of different types of reasons for sick leave. We are in the process of analyzing these register-based datasets (with 407 different diagnoses given for sick leave) for upcoming publications. For this study, we investigated if being on sick leave (in general) was associated with impairment in this study. In the limitations section we added that:” Physical and psychological health are closely connected, and the study did not clarify the potentially differential impact of physical health illnesses compared to mental health diagnoses.”

**5.** **Results**. It looks like comorbidity was addressed in the analyses, but where there differences in the overall severity of patient groups?

***Reply***: The study analyzed differences in impairment between groups but did not include measures of symptom severity. To accommodate your comment, we have added the following to the discussion: “Another limitation of this study is that it did not include measures of symptom severity. Research from the same clinic as subsample 2 has shown that symptom severity is associated with health-related quality of life and that patients with personality disorders reported the lowest quality of life followed by trauma-related disorders, depression, and anxiety, while patients with ADHD reported the best quality of life [64].”

**6.** Pg 15, 1st paragraph. I’m not convinced this study is using the most “heterogenous samples” for EFA and CFA compared to other studies. As mentioned above, the EFA was conducted on sample with mild symptoms and CFA on moderate to severe symptoms. By analyzing these separately, the individual samples are less heterogenous than if the authors would have combined the two samples and conducted EFA on one ½ of the sample and CFA on the other ½. This would have allowed for the modeling of symptom range from mild to severe in both the EFA and CFA, rather than mild in EFA and mod/severe in the CFA.

***Reply*:** Thank you for your comment. We chose to label the samples as heterogenous as there was a multitude of diagnoses and presenting problems included. As mentioned, we have revised the analyses by combining the subsamples covering the full spectrum of symptom severity.

**7. General discussion comment**. Please add issue related to the impact of physical health illnesses impacting the sick leave group. We cannot assume that it is only the mental health diagnoses that are driving the results in the sick leave group.

***Reply***: We have added the following to the discussion: “Physical and psychological health are closely connected, and the study did not clarify the potentially differential impact of physical health illnesses compared to mental health diagnoses.”
